# Early Detection of Cardiovascular Risk Factors and Definition of Psychosocial Profile in Women Through a Systematic Approach: The Monzino Women Heart Center's Experience

**DOI:** 10.3389/fcvm.2022.844563

**Published:** 2022-03-08

**Authors:** Sebastiano Gili, Mattia Giuliani, Giulia Santagostino Baldi, Giovanni Teruzzi, Gabriella Pravettoni, Piero Montorsi, Daniela Trabattoni

**Affiliations:** ^1^Centro Cardiologico Monzino, Istituto di Ricovero e Cura a Carattere Scientifico (IRCCS), Milan, Italy; ^2^Applied Research Division for Cognitive and Psychological Science, Istituto Europeo di Oncologia (IEO), European Institute of Oncology IRCCS, Milan, Italy; ^3^Department of Clinical Sciences and Community Health, University of Milan, Milan, Italy

**Keywords:** gender medicine, women heart disease, cardiovascular prevention, psychological assessment, anxiety and stress

## Abstract

**Introduction:**

Cardiovascular (CV) disease is the leading mortality cause among women, yet an alarming misrepresentation of women in CV studies and a low awareness of the impact of CV among women still persist to date. The Monzino Women Heart Center has been established as a clinical and research program dedicated to primary prevention of CV disease in women.

**Methods:**

Patients aged between 35 and 60 years and with no history of CV disease underwent a comprehensive evaluation including a cardiologic outpatient visit with electrocardiogram, individual CV risk calculation, first-level cardiovascular examinations and a psychological assessment.

**Results:**

A total of 635 women, with a mean age of 52.2 ± 6.4 participated to the project on a voluntary basis during the period January 2017–August 2021. Included patients had a high level of education (40.4% with a graduate or postgraduate university degree), the majority of them, in a stable couple and with children, were actively working. More than half of the patients performed physical activity on a regular basis. Prevalence of traditional CV risk factors were family history (70.2%), hypertension (46%), hypercholesterolemia (22%) and diabetes (14%). Early or premature menopause was reported by 17.7% of the patients, gestational hypertension and diabetes by 4.96 and 1.7%, respectively. Symptoms of depression were reported by 27%; nearly 36% of the participants rated high score of state anxiety and 41% of trait anxiety. Nearly 69% of the participants showed moderate-to-high perceived stress. The mean value of perceived general self-efficacy was moderate (mean = 28.78, SD = 4.69).

**Conclusion:**

A CV prevention program dedicated to women can help identifying a considerable number of patients with risk factors for whom early interventions can help reducing the risk of developing CV disease. Psychological assessment might unmask depression or anxiety disorders, which might have a potential long-terme detrimental effect on CV health.

## Introduction

Cardiovascular disease is the leading cause of mortality in women, accounting for 35% of deaths worldwide in 2019 and for 37.7% of total deaths in Italy in 2018 (1, 2). Despite prevention programs and educational campaigns conducted over the last two decades, the awareness of the impact of cardiovascular disease on mortality among women declined from 64.8% in 2009 to 43.7% in 2019 (3) in the United States, particularly in younger and low-educated ones (4). There is a glaring and alarming gender disparity in health literature, particularly regarding cardiovascular aspects: women are under-represented in clinical trials participation, sex-specific data is under-reported and, in general, women represented <39% of cardiovascular clinical trial participants between 2010 and 2017 (5). All these factors considerably limited the potential for evaluating the safety and efficacy of therapies specifically addressed to women, identifying sex-specific differences in cardiovascular outcomes and developing new sex-related strategies in the management and prevention of cardiovascular disease in women (1).

In order to tackle these issues, Monzino Women's Heart Center has been created in early 2017 as the first established center in Italy fully dedicated to the prevention, detection, and treatment of heart disease in women and to the development of a research program on this topic. The main area of focus of the program is the primary prevention and early diagnosis of heart and atherosclerotic vascular disease in women, through outpatient visits and non-invasive cardiovascular examinations and educational initiatives dedicated to general population. Program activities are run by a specifically trained multidisciplinary team (physicians, nurse practitioners, dieticians, medical technicians and an exercise physiologist). The “Monzino Women” program can be accessed through the National Health System and is completely free for all participants and to date represents the only structured center fully dedicated to the study and treatment of cardiovascular disease in women in Italy.

Aim of the present paper is to present “Monzino Women” to a wider scientific audience as a reproducible organizational system for the primary prevention of cardiovascular disease in women and to present data collected so far.

## Methods

**Figure 1:**
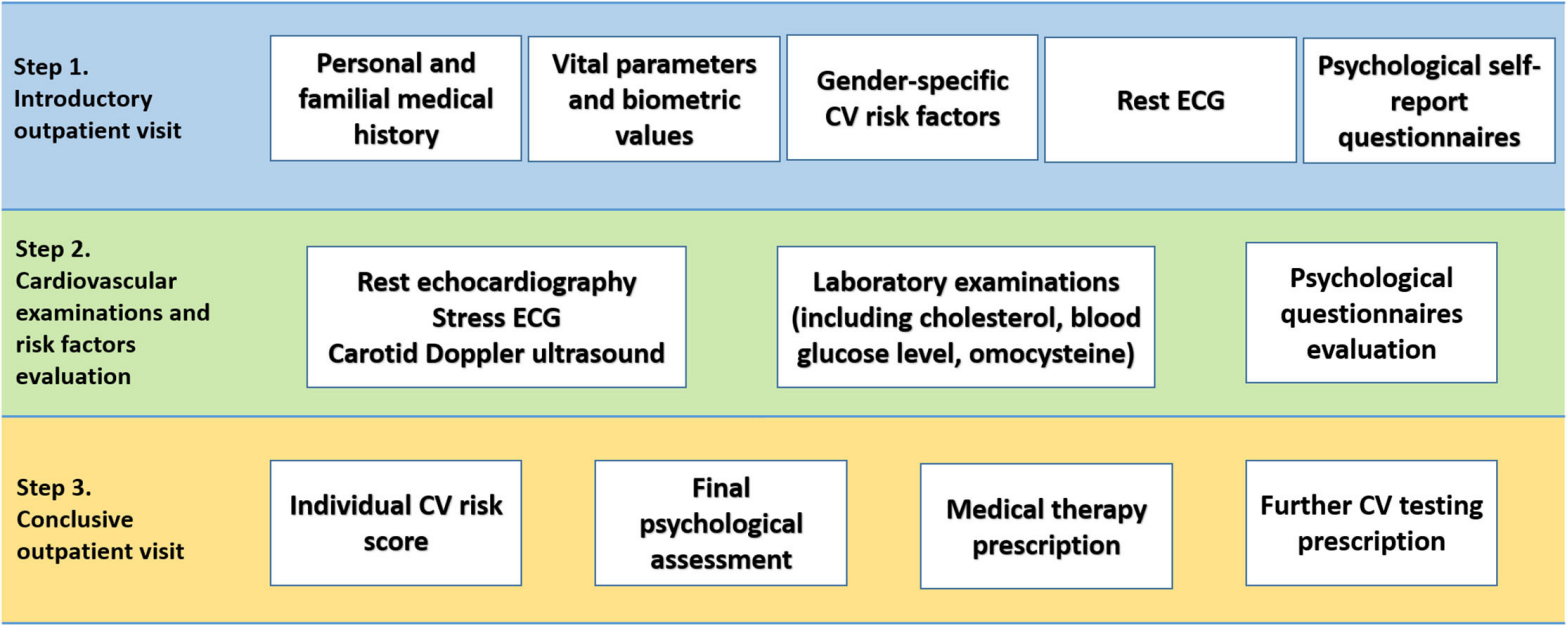
Organizational workflow of Monzino Women's Heart Center.

All consecutive patients participating on a voluntary basis from January 2017 to August 2021 at the “Monzino Women's Heart Center” project for primary prevention and early diagnosis of cardiovascular disease were included in the present analysis. Patients needed to be aged between 35 and 60 years and to present no history of overt cardiovascular disease. A schematic representation of the organization of Monzino Women's Heart Center outpatient program is provided in Figure 1. Patients underwent a comprehensive evaluation based on a standardized protocol, including a cardiologic outpatient visit with electrocardiogram, a psychological assessment, a transthoracic echocardiogram, an ECG exercise stress test, a carotid Doppler ultrasound and laboratory tests.

Baseline clinical data were collected for all patients, including biometric data, vital parameters including arterial blood pressure, traditional cardiovascular risk factors (i.e., smoking habit, familiar history of cardiovascular disease, hypertension, dyslipidemia, diabetes mellitus, obesity/overweight), “women-specific” cardiovascular risk factors (i.e., early or premature menopause, gestational diabetes and/or hypertension, eclampsia, pre-term delivery) and past medical history, with a particular focus on endocrine, autoimmune and gynecological disorders. Drugs prescription history was as well-documented.

A full transthoracic echocardiogram with colorDoppler was conducted for each patients based on current European Association of Cardiovascular Imaging recommendations (6). Stress ECG was executed on a stationary bike with a workload increase of 25W every 2 min; the test was defined “maximal” if heart rate at peak reached 85% of age-predicted maximal heart rate (APMHR; calculated as: 220—age); patients were demanded in any case to exercise until their maximum effort and were not stopped solely for reaching 85% of APMHR (7). A carotid Doppler ultrasound was performed in order to measure intimal-media thickness and to assess the presence of subclinical atherosclerosis (8). Laboratory tests included, but were not limited to, evaluation of lipid profile, fasting blood glucose level, homocisteine.

Every patient was finally given a conclusive summary comprising the estimated risk of cardiovascular events calculated with the Progetto Cuore score (9) and an extensive consultation about lifestyle changes required to reduce cardiovascular risk. In case of abnormal findings, further diagnostic examinations or therapeutic interventions were prescribed in conformity with current practice.

All patients received a self-report psychological battery which assessed several domains: symptoms of depression, anxiety, perceived distress, perceived social support, personality traits, general self-efficacy, and hostility. The psychologist, a member of the team, rated the questionnaires and returns to the participants a report of their results. Whenever the questionnaires report clinically relevant results for depression, anxiety and distress, the participants are invited to a free interview with the psychologist and, if necessary, to set up specific psychological support.

### Psychological Questionnaires

The participants completed the following self-report and validated questionnaires.

- Beck Depression Inventory—II (BDI-II): the BDI-II is a self-report questionnaire with 21 items that assess severity of depressive symptoms in adults and adolescents (10). Each item of the BDI-II is rated 0–3 by the patient. The summed ratings across all items yield a total score. Based on this total score, severity of depressive symptoms is ranked into four levels: 0–13 (minimal depressive symptoms), 14–19 (mild depressive symptoms), 20–28 (moderate depressive symptoms) and 29–63 (severe depressive symptoms). The Italian version of the BDI-II used in this study has shown high internal consistency (Cronbach α =0.80), good convergent validity with the Depression Questionnaire (11), and good test-retest reliability after 1 month (*r* = 0.76) (12).
- State-Trait Anxiety Inventory—Y form (STAI-Y): The STAI-Y is a widely used self-report questionnaire that measures state and trait anxiety (13). The STAI-Y includes 20 items for assessing the level of state anxiety. State anxiety is anxiety felt at the moment of the assessment and is thought to be temporary and context-dependent. Twenty further items on the STAI-Y assess the level of trait anxiety. Trait anxiety is anxiety usually felt in everyday life and is thought to be stable and context-independent. All items are evaluated on a 4-point scale with a total score that ranges from 20 to 80 for both the state and trait anxiety subscales. The STAI-Y does not have a cut-off score, but higher scores suggest more significant anxiety. The STAI-Y has shown high internal consistency (Cronbach α = 0.93 for state anxiety and Cronbach α = 0.90 for trait anxiety). The STAI-Y Italian version was used in the present study (14).
- Perceived Stress Scale (PSS): the PSS is the most widely used self-report questionnaire for measuring the perception of stress (15). The items evaluate the frequency of feelings and thoughts related to distress perception during the last month. The scores range from 0 to 40, with higher scores indicating a higher level of perceived distress. The score interpretation is based upon 3 values categories: 0–13 (i.e., low stress), 14–26 (i.e., moderate stress) and 27–40 (i.e., high stress). The Italian version of the PSS was used (16).
- Multidimensional Scale of Perceived Social Support (MSPSS): the MSPSS is a 12-items questionnaire designed to measure perceptions of support from 3 sources: Family, Friends, and a Significant Other. The total score ranges from 1 to 7, with 3 interpretation categories: from 1 to 2.9 (i.e., perception of low social support), from 3 to 5 (i.e., perception of moderate social support) and from 5.1 to 7 (i.e., perception of high social support). The Italian version of the MSPSS was used (17).
- Distressed Personality Scale (DS-14): the DS-14 is a brief self-report questionnaire which is worldwide used to evaluate type D personality traits: negative affectivity (NA) and social inhibition (SI) (18). The DS-14 comprises 14 items, each evaluated on a scale between 0 (i.e., false) and 4 (i.e., true). It provides two separate score for NA and SI, each range from 0 to 28. A cut-off score of ≥10 means the presence of a maladaptive personality trait.
- General Self-Efficacy (GSE): the GSE has become a widely used instrument for measuring general self-efficacy. The GSE assesses “a broad and stable sense of personal competence to deal effectively with a variety of stressful situations.” It consists of 10 items that are rated on a scale from 1 (“not at all true”) to 4 (“exactly true”). The GSE sum score is calculated by summing the item scores, and ranges between 10 (lowest GSE) and 40 (highest GSE). The Italian version of the GSE was used (19).
- Buss-Durkee Hostility Inventory (BDHI): the BDHI is a 75 true/false items questionnaire which assesses a general negative, distrustful attitude toward others, which is accompanied by hidden negative feelings (hostility) (20). The questionnaires provides several hostility subdomains: assault, indirect hostility, irritability, negativism, resentment, suspicion, verbal hostility, and guilt. Scores higher than 60 are considered to be clinically relevant.

### Statistical Analysis

Continuous variables were expressed as mean ± standard, qualitative variables were reported as number and percentage. Normality was assessed through the Shapiro-Wilk test for normality and differences among groups were tested using non-parametric Mann-Whitney *U*-test. Missing values were managed with a complete case analysis, i.e., by simply omitting missing data from analyses. A two-sided *p*-value of 0.5 was set as statistically significant. All analyses were performed using Jamovi version 1.6.21.0.

## Results

**Figure 2:**
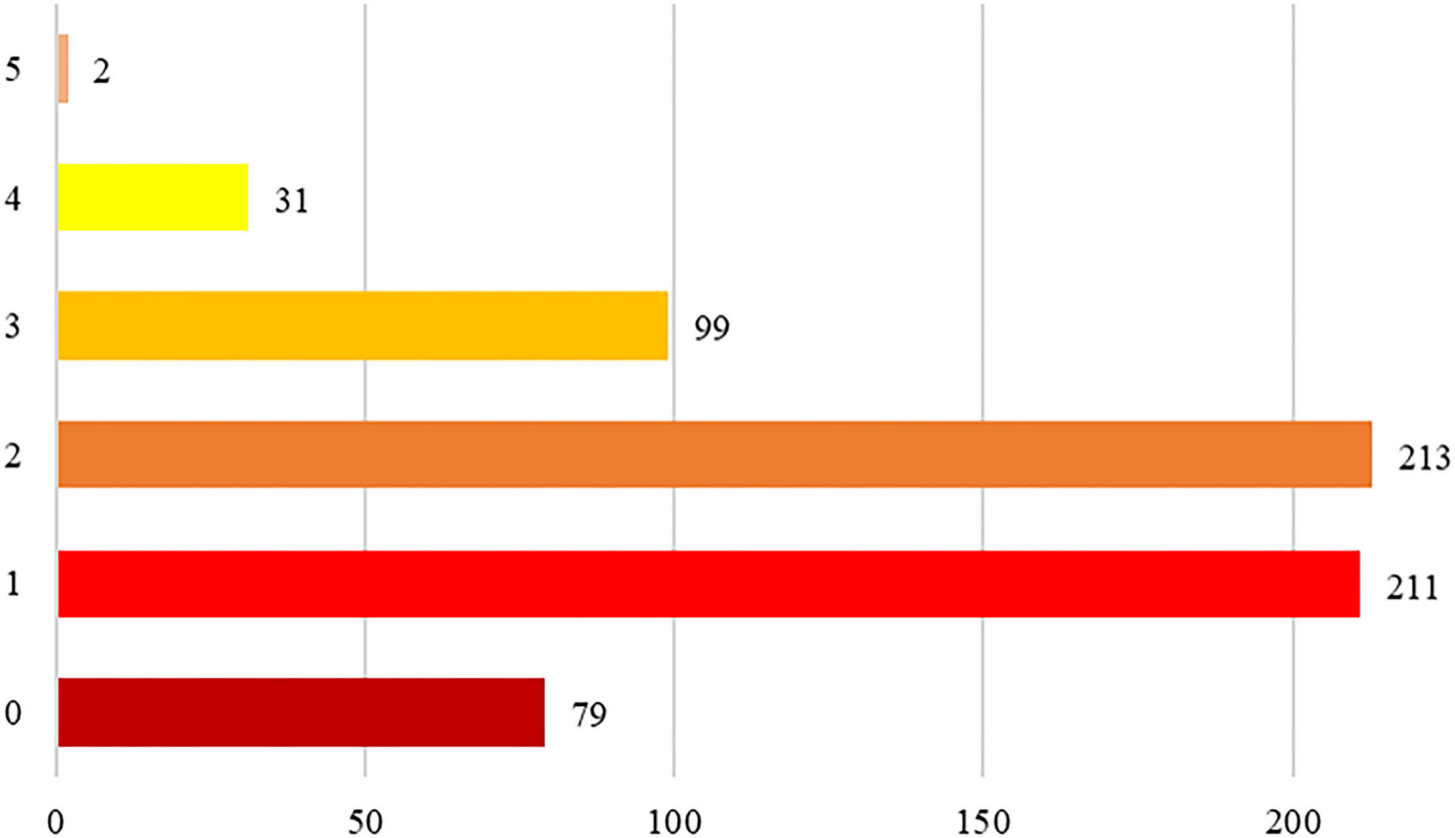
Number of traditional cardiovascular risk factors per paper in the study population.

A total of 635 women, with a mean age of 52.2 ± 6.4 years, took part to the project from January 2017 to August 2021 and were included in the present analysis. Baseline characteristics of study population are shown in Tables 1–3; in general, patients had a high level of education (40.4% with a graduate or postgraduate degree), the majority of them was married or living in a stable couple and had children, and almost all of them were actively working. At least one traditional risk factor was reported by 435 (64%) patients, with most patients reporting more than one (Figure 2). The most frequent “traditional” cardiovascular risk factor was family history of cardiovascular disease (70.2%); among modifiable traditional risk factors, hypertension was the more prevalent (46%), followed by hypercholesterolemia (22%), active cigarette smoking (17%), past smokers (11%) and diabetes (14%). More than half of the patients reported to perform physical activity on a regular basis. Mean plasmatic level of LDL and HDL were 125 and 63 mg/dl, respectively. Early or premature menopause was reported by 17.7% of the patients, gestational hypertension and diabetes by 4.9 and 1.7% patients, respectively. Estroprogestinic or progesterone-only therapy for birth control was taken by one quarter of study population.

**Table 1:** Sociodemographic and baseline features.

	***N* = 635**
Age (years)^∫^	52.21 ± 6.42
**Education**	
Elementary school	0 (0)
Intermediate school	44 (6.9)
High school	335 (52.8)
University	223 (35.2)
Post-university (i.e., PhD)	33 (5.2)
**Marital status**	
Single	85 (13.4)
In a relationship	48 (7.6)
Married	441 (69.5)
Divorced	54 (8.5)
Widow	7 (1.1)
**Employment status**	
Employed	618 (97.3)
**Offspring**	
0	176 (28.1)
1	295 (47.0)
2	132 (21.1)
3+	24 (3.8)

*Values are expressed as number (%), excepted for ^∫^, which is reported as mean ± standard deviation*.

**Table 2:** “Classic” cardiovascular risk factors.

	***N* = 635**
Familiar history of cardiovascular disease	446 (70.2)
**Smoking habit**	
Smoker	108 (17.0)
Past smoker	71 (11.2)
**Alcohol consumption**	
Occasional drinker	110 (17.3)
Regular drinker	10 (1.6)
**Physical activity**	
Sedentary	278 (43.8)
Occasional physical activity	5 (0.6)
Regular physical activity	353 (55.6)
Hypertension	291 (45.8)
Diabetes	91 (14.3)
Overweight	188 (41.9)
Hypercolesterolaemia	139 (21.9)

*Values are expressed as number (%)*.

**Table 3:** Women specific cardiovascular risk factors.

	***N* = 635**
Early or premature menopause	53 (17.7)
Menopausal hormone replacement therapy	48 (7.6)
Polycystic ovary syndrome	17 (2.7)
Preterm delivery	21 (3.3)
Miscarriage	199 (31.3)
Eclampsia	10 (1.6)
Gestational hypertension	31 (4.9)
Gestational diabetes	11 (1.7)
Systemic inflammatory and autoimmune disorsers	10 (1.6)

*Values are expressed as number (%)*.

Following the completion of the clinical evaluation, pharmacological therapy was prescribed to 143 (22.5%) patients, with statins being the most frequently prescribed drugs (13.7%), followed by ACE-inhibitors/angiotensin II receptor blockers (6.8%) and beta-blockers (4.6%). A limited number of patients were prescribed benzodiazepines or anti-depressants (serotonin and norepinephrine reuptake inhibitor or selective serotonin reuptake inhibitors), also based on the results of psychological evaluation (Supplementary Table 1). Further clinical investigations were carried out in 22 (3.5%) patients, including coronary computed tomography (*n* = 6), coronary angiography (*n* = 13), percutaneous coronary intervention (*n* = 2) and patent foramen ovale percutaneous closure (n=1).

### Psychological Assessment

At the psychological self-report battery symptoms of depression were reported by nearly 27% of the participants, most of them rated as mild (15.7%). Nearly 36% of the participants rated high score of state anxiety during the self-report battery administration and 41% showed high trait anxiety. Nearly 69% of the participants showed moderate-to-high perceived stress. Most of the participants declared high level of perceived social support from a significant other (63.6%), moderate social support from friends (60.7%) and nearly the same percentages for both moderate (46.7%) and high (48.9%) social support from family. More than the half of the participants showed high negative affectivity (54.2%) and 38% rated high social inhibition. The mean value of perceived general self-efficacy was moderate (mean = 28.78, SD = 4.69). The hostility scores were above the clinical cutoff only for a few participants, with “resentments” being the more prevalent hostility subdomain (20%). All these results are showed in Table 4. Finally, as shown in Supplementary Table 2 the psychological characteristics of those patients who received a pharmacological prescription did not differed from those who did not receive it. As expected, those participants who were asked to undergo an in-depth psychological assessment (*n* = 45; 7%) showed general worse psychological symptoms than those who were not. In particular, aside from depression, anxiety and distress, participants also showed lower perceived social support, maladaptive personality traits, lower self-efficacy and higher levels of hostility (Supplementary Table 3).

**Table 4:** Psychological characteristics of the study population.

	***N* = 635**
**Symptoms of depression (BDI-II)**	
Low	83 (15.7)
Moderate	47 (8.9)
Severe	14 (2.7)
State anxiety (STAI-S)	190 (35.9)
Trait anxiety (STAI-T)	215 (41.0)
**Perceived distress (PSS)**	
Low	164 (31.2)
Moderate	317 (60.3)
Severe	45 (8.5)
**Perceived social support (MSPSS)**	
**Significant other**	
Low	8 (1.5)
Moderate	182 (34.9)
High	331 (63.6)
**Friends**	
Low	17 (3.3)
Moderate	316 (60.7)
High	188 (36.0)
**Family**	
Low	23 (4.4)
Moderate	243 (46.7)
High	255 (48.9)
**Distressed Personality (DS-14)**	
Negative affectivity	279 (54.2)
Social inhibition	197 (38.2)
General self-efficacy (GSE) ^∫^	28.78 ± 4.69
**Hostility (BDHI)**	
Assault	48 (9.2)
Indirect hostility	51 (9.8)
Irritability	27 (5.2)
Negativism	43 (8.3)
Resentment	104 (20.0)
Suspicion	93 (17.9)
Verbal hostility	48 (9.2)
Guilt	90 (17.3)

*Values are expressed as number (%), excepted for ^∫^, which is reported as mean ± standard deviation. BDI-II, Beck Depression Inventory II; STAI, State-Trait Anxiety Inventory; PSS, perceived stress scale; MSPSS, Multidimensional Scale of Perceived Social Support; DS-14, Distressed Personality Scale; BDHI, Buss-Durkee Hostility Inventory*.

## Discussion

The present report shows that a cardiovascular prevention program specifically dedicated to women can help identifying a considerable number of patients with cardiovascular risk factors for whom early interventions—through lifestyle changes and medical therapy—can help reducing the risk of developing cardiovascular disease. In particular, we have documented that through such a structured approach, a relevant number of patients (almost one out of four) needing disease-altering medical therapy could be identified. Moreover, the thorough assessment of individual psychological status might unmask depression or anxiety disorders, which might have a potential long-term detrimental effect on the cardiovascular health of participating patients.

In the last years, the renewed interest on female-specific cardiovascular disease risk factors (21) contributed to enhance gender-based cardiovascular research. Compared to men, women present a peculiar set of psycho-socio-economic and cultural factors that might contribute to the development, maintenance and aggravation of cardiovascular disease and that, to date, are still largely not taken into account in clinical practice. Beyond the traditional cardiovascular risk factors, additional women-specific risk factors, such as early or premature menopause, pregnancy complications (gestational diabetes, hypertension onset during pregnancy, eclampsia, preterm delivery), polycystic ovary syndrome, systemic and autoimmune disorders, breast cancer and subsequent chemio- and radiation-therapies have to be considered in the cardiovascular risk assessment of women (1, 21). Of note, European Society Guidelines on cardiovascular prevention have acknowledged and integrated, in their latest version, some of the gender-specific risk factors described above. They still failed, however, to officially define a gender-specific risk evaluation protocol fully considering all the peculiar aspects relating to each gender (22). During the course of our activity, we identified a relevant number of patients with traditional and gender-specific cardiovascular risk factors, who could mostly benefit from early detection and treatment of such conditions in order to improve women's cardiovascular health and reduce their mortality (1). The importance of developing a program for the management of cardiovascular disease for women is of particular importance considering that the treatment of women's heart disease was based—until recently—on medical research biased by a disproportioned balance between men and women (23). It is in fact well-documented that specific issues related to female gender have been historically underrepresented in research studies, so that little is known about the differences in biomarkers profile in women and men, the impact of pregnancy and its complications on subsequent cardiovascular diseases in the newborn and mother, the social determinants of health and their impact on cardiovascular outcomes in women and men, the relationship between non-traditional CV risk factors, such as systemic autoimmune disorders and the effects of psychological factors such as high anxiety and life stress on chest pain symptoms in women. In recent years these issues have been recognized as unmet needs in medicine, so that the American Heart Association (AHA) launched the Go Red for Women initiative in 2004, with the aim of developing an integrated approach to the care of women with heart disease (24), sparking the emergence of several centers of excellence dedicated to women's health across the United States. In most of the American Women's Heart Centers a leading role is played in identifying female-pattern heart disease, developing new diagnostic tools and advancing specialized care for women (25). The general principles regulating such centers are similar to ours, with the main focus being on early diagnosis and prevention of cardiovascular disease, clinical and translational research and educational initiatives oriented at increasing patients' and physicians' awareness. Some differences among these centers may be observed due to differences in local settings and expertise: for example, some centers are more focused on neurodegenerative disease, some others on obstetrical/gynecological related cardiovascular disease (25). Our center has been created in a single specialty hospital for cardiovascular disease, nonetheless we involved a psychological team with the aim of working on the connections between psychological wellbeing and cardiovascular disease.

A critical step for improving cardiovascular health in women is to increase their awareness regarding their actual risk of developing cardiovascular disease and the factors and actions increasing or reducing such risk (25). It is on this view that one of the core principles of Monzino Women's Heart Center is the divulgation and education of the potential risks of women to develop cardiovascular disease. Mosca et al. demonstrated that women's heart disease awareness is increasing in the United States, with the number of women aware that heart disease is the leading cause of death nearly doubling in the last 15 years, but that this knowledge still lags in minorities and younger women (26). From this point of view, a critical aspect emerging from our analysis is the high level of education of included patients, which highlights how some less educated patients might still be excluded and not reached by our initiative.

Also depression, anxiety and stress, which are recognized risk factors for cardiovascular disease (27–33) are more frequently observed in women than men (34–36). The relationship between depression, depressive symptoms and cardiovascular diseases is a well-known issue. In 2008, the AHA published a consensus paper in which clearly stated that depression acts as an independent risk factor for cardiovascular diseases, in particular for coronary artery disease and myocardial infarction (37). Other studies have shown that depression can worsen the prognosis (i.e., augment the number of re-hospitalization, increase the length of hospital stay and increase the all-cause mortality of patients) of patients with cardiovascular diseases (38–40). Nevertheless, also anxiety and stress were found to play a significant role in cardiovascular diseases. Several studies found that anxiety disorders (and symptoms) and stress were related with the onset and progression of cardiovascular diseases, and have been linked to adverse cardiovascular outcomes, including mortality (41). Similar evidence was found for stress and cardiovascular diseases. The relationship between depression, stress, anxiety and cardiovascular diseases may have multifaceted aspects, involving both physiological processes (i.e., autonomic dysfunction, inflammation, endothelial dysfunction, changes in platelet aggregation, etc.) and health behavior (i.e., self-care motivation reduction, disease self-management, drop-outs, etc.) (42, 43).

Finally, it has to be underlined that a relevant part of our effort has been dedicated to design a “reproducible” organizational system, which can be replicated in other areas of our country to improve the treatment of cardiovascular disease in women. For this reason, we designed an organizational model, which is defined by clearly delignated diagnostic steps, is run by certified professional figures acting in their respective area of expertise and has clearly defined objectives: quantification of traditional CV risk factor, CV risk score calculation, prescription of guideline-recommended treatments and examinations and evaluation and documentation of emerging gender-related risk factors. Obviously further challenges are ahead after this initial report, in particular the evaluation of the effectiveness of such an intensive approach in primary CV prevention.

The present study has several limitations: sample size is limited, which prevents us from generalizing our findings; the study population is formed by women participating on a voluntary basis upon spontaneous presentation, which introduces a selection bias, as can be seen in particular by the high education level of our patients.

## Conclusions

Women are still labeled as a special population in many guidelines relating to CVD. Particularly when presenting with acute coronary syndromes, they tend to experience worse outcomes, especially at younger ages (44), at least partly because they are less likely to receive guideline-recommended treatments and diagnostic or invasive evaluations (45, 46). It's a common belief that women are better at looking after their health than men. But when it comes to heart health, research shows that many women don't. The Monzino Women Heart Center, which has been extensively presented in the current document, has been designed in order to contribute to fill the gender gaps and issues still present in cardiovascular medicine and as a pilot project which could be replicated and validated in broader settings.

## Data Availability Statement

The datasets presented in this study can be found in online repositories. The names of the repository/repositories and accession number(s) can be found below: ZENODO 10.5281/zenodo.5807307.

## Ethics Statement

The studies involving human participants were reviewed and approved by Centro Cardiologico Monzino EC. The patients/participants provided their written informed consent to participate in this study.

## Author Contributions

DT and MG conception and design of the study. GS and DT organized the database. SG and MG performed the statistical analysis. MG wrote the first draft of the manuscript. GS, DT, and GT wrote sections of the manuscript. All authors contributed to the article and approved the submitted version.

## Conflict of Interest

The authors declare that the research was conducted in the absence of any commercial or financial relationships that could be construed as a potential conflict of interest.

## Publisher's Note

All claims expressed in this article are solely those of the authors and do not necessarily represent those of their affiliated organizations, or those of the publisher, the editors and the reviewers. Any product that may be evaluated in this article, or claim that may be made by its manufacturer, is not guaranteed or endorsed by the publisher.
