# Supplementary material for: Early Detection of Cardiovascular Risk Factors and Definition of Psychosocial Profile in Women Through a Systematic Approach: The Monzino Women Heart Center's Experience
Source: Front Cardiovasc Med. 2022 Mar 8;9:844563. doi: 10.3389/fcvm.2022.844563 (PMC8957226; doi:10.3389/fcvm.2022.844563)
Supplement: Supplementary file 1 [file Data_Sheet_1.docx]

**Supplementary Table 1.** Prescribed drugs after conclusive evaluation at Monzino Women’s Heart Center

|  |  | |
| --- | --- | --- |
| **Drug** | ***N*** | ***%*** |
| Statins | 87 | 13.7 |
| ACE-inbhitors/ARB | 43 | 6.8 |
| Beta blockers | 29 | 4.6 |
| Aspirin | 15 | 2.4 |
| Folate | 12 | 1.9 |
| Vitamin D supplementation | 10 | 1.6 |
| Benzodiazepine/SSRI/SNRI | 13 | 2.1 |
| Ezetimibe | 10 | 1.6 |
| Other drugs | 5 | 0.8 |
| ACE, angiotensin converting enzyme; ARB, angiotensin II receptor blockers;  SNRI, serotonin and norepinephrine reuptake inhibitor;  SSRI, Selective serotonin reuptake inhibitors  Other drugs include direct oral anticoagulant, antiarrhythmic drugs, antidiabetic drugs | | |

|  | **W/o prescription**  **(n = 492)** | | **W/ prescription**  **(n = 143)** | | **Mann-Whitney**  **(z score)** | **p-value** |
| --- | --- | --- | --- | --- | --- | --- |
|  | *Mean* | *SD* | *Mean* | *SD* |  |  |
| Symptom of Depression (BDI-II) | 10.13 | 7.53 | 10.34 | 7.66 | -0.38 | 0.704 |
| State Anxiety (STAI-S) | 41.76 | 10.05 | 41.70 | 9.89 | -0.081 | 0.935 |
| Trait Anxiety (STAI-T) | 42.27 | 9.69 | 42.76 | 8.26 | -0.57 | 0.569 |
| Perceived distress (PSS) | 17.08 | 6.86 | 16.80 | 6.95 | -0.268 | 0.789 |
| Perceived Social Support. Significant other  (MSPSS) | 5.71 | 1.18 | 5.64 | 1.25 | -0.234 | 0.815 |
| Perceived Social Support, Family  (MSPSS) | 5.26 | 1.58 | 5.33 | 1.34 | -0.938 | 0.348 |
| Perceived Social Support, Friends  (MSPSS) | 5.06 | 1.51 | 5.05 | 1.34 | -0.744 | 0.457 |
| Perceived Social Support, Total score  (MSPSS) | 4.73 | 2.64 | 4.78 | 2.01 | -0.132 | 0.895 |
| Distressed Personality. Negative Affectivity (DS-14) | 11.19 | 6.50 | 11.34 | 7.26 | -0.217 | 0.829 |
| Distressed Personality. Social Inhibition  (DS-14) | 8.03 | 6.00 | 8.06 | 6.00 | -0.091 | 0.927 |
| General Self-Efficacy (GSE) | 28.64 | 4.66 | 29.33 | 4.78 | -1.086 | 0.277 |
| Assault (BDHI) | 47.42 | 7.31 | 46.50 | 6.36 | -1.45 | 0.147 |
| Indirect Hostility (BDHI) | 46.65 | 10.37 | 47.61 | 11.68 | -1.199 | 0.231 |
| Irritability (BDHI) | 43.95 | 8.55 | 45.72 | 9.33 | -2.102 | **0.036** |
| Negativism (BDHI) | 47.23 | 10.85 | 46.55 | 11.63 | -0.584 | 0.559 |
| Resentment (BDHI) | 49.28 | 10.63 | 48.56 | 10.94 | -0.553 | 0.580 |
| Suspicion (BDHI) | 48.63 | 9.74 | 47.38 | 9.90 | -1.083 | 0.279 |
| Verbal Hostility (BDHI) | 45.58 | 10.10 | 45.94 | 11.03 | -0.169 | 0.866 |
| Guilt (BDHI) | 49.72 | 8.76 | 51.17 | 10.52 | -1.22 | 0.222 |

**Supplementary Table 2.** Psychological characteristics stratified for pharmacological prescriptions.

**Supplementary Table 3**. Psychological characteristics stratified for those participants who were asked for an in-depth psychological assessment and those who did not.

|  | **No psychological support** | | **Psichological support** | | **Mann-Whitney**  **(z score)** | **p-value** |
| --- | --- | --- | --- | --- | --- | --- |
|  | *Mean* | *SD* | *Mean* | *SD* |  |  |
| Symptom of Depression (BDI-II) | 8.68 | 5.69 | 25.08 | 6.41 | -10.71 | **<0.001** |
| State Anxiety (STAI-S) | 40.41 | 8.98 | 55.15 | 9.97 | -8.13 | **<0.001** |
| Trait Anxiety (STAI-T) | 40.83 | 8.23 | 57.65 | 6.04 | -10.38 | **<0.001** |
| Perceived distress (PSS) | 16.03 | 6.25 | 26.94 | 4.52 | -9.68 | **<0.001** |
| Perceived Social Support. Significant other (MSPSS) | 5.76 | 1.17 | 5.05 | 1.19 | -3.94 | **<0.001** |
| Perceived Social Support. Family (MSPSS) | 5.38 | 1.49 | 4.16 | 1.54 | -5.23 | **<0.001** |
| Perceived Social Support. Friends (MSPSS) | 5.13 | 1.47 | 4.29 | 1.35 | -3.99 | **<0.001** |
| Perceived Social Support. Total score (MSPSS) | 4.82 | 2.61 | 3.95 | 1.06 | -4.05 | **<0.001** |
| Distressed Personality. Negative Affectivity (DS-14) | 10.30 | 6.10 | 20.76 | 4.29 | -9.14 | **<0.001** |
| Distressed Personality. Social Inhibition (DS-14) | 7.69 | 5.85 | 11.67 | 6.38 | -4.09 | **<0.001** |
| General Self-Efficacy (GSE) | 29.17 | 4.48 | 24.83 | 4.99 | -5.74 | **<0.001** |
| Assault (BDHI) | 46.95 | 6.80 | 50.13 | 9.53 | -1.80 | 0.071 |
| Indirect Hostility (BDHI) | 46.28 | 10.41 | 52.70 | 11.42 | -3.64 | **<0.001** |
| Irritability (BDHI) | 43.72 | 8.58 | 50.39 | 8.05 | -4.86 | **<0.001** |
| Negativism (BDHI) | 46.68 | 10.96 | 51.33 | 10.68 | -2.80 | **0.005** |
| Resentment (BDHI) | 48.19 | 10.46 | 58.80 | 7.85 | -6.83 | **<0.001** |
| Suspicion (BDHI) | 47.90 | 9.62 | 53.28 | 10.08 | -3.39 | **<0.001** |
| Verbal Hostility (BDHI) | 45.41 | 10.14 | 48.11 | 11.45 | -1.48 | 0.140 |
| Guilt (BDHI) | 49.31 | 8.97 | 57.20 | 7.84 | -5.52 | **<0.001** |
